# Progesterone: A Unique Hormone with Immunomodulatory Roles in Pregnancy

**DOI:** 10.3390/ijms23031333

**Published:** 2022-01-25

**Authors:** Raj Raghupathy, Julia Szekeres-Bartho

**Affiliations:** 1 Department of Microbiology, Faculty of Medicine, Kuwait University, Safat 13110, Kuwait; 2 Department of Medical Biology and Central Electron Microscope Laboratory, Medical School, University of Pécs, 7624 Pécs, Hungary; szekeres.julia@pte.hu; 3 National Laboratory for Human Reproduction, University of Pécs, 7624 Pécs, Hungary; 4 János Szentágothai Research Centre, University of Pécs, 7624 Pécs, Hungary; 5 MTA-PTE Human Reproduction Scientific Research Group, University of Pécs, 7624 Pécs, Hungary

**Keywords:** progesterone, progestogens, dydrogesterone, pregnancy, pregnancy complications, immunomodulation, cytokines

## Abstract

Progesterone is well known for its numerous endocrinologic roles in pregnancy but is also endowed with fascinating immunomodulatory capabilities. It can downregulate the induction of inflammatory reactions, the activation of immune cells and the production of cytokines, which are critical mediators of immune responses. These features appear to be critical to the success of pregnancy, given the ability of maternal immune reactivity to interfere with pregnancy and to contribute to several pregnancy complications. This review summarizes the contribution of maternal immune effectors in general, and cytokines in particular, to pregnancy complications such as recurrent miscarriage, pre-eclampsia and preterm labor; it describes the promise offered by supplementation with progesterone and the oral progestogen dydrogesterone, as well as the progesterone-induced blocking factor in the prevention and/or treatment of these serious complications.

## 1. Introduction

Progesterone plays an impressive range of extremely important endocrinologic roles such as stimulating the growth of blood vessels that supply the endometrium, stimulating the endometrium to secrete nutrients that nurture the early embryo, preparing the uterine lining for implantation of the embryo and sustaining the endometrium throughout pregnancy. Later during gestation, by activating progesterone receptor B, this hormone stimulates the development of the mammary gland and fortifies the pelvis in preparation for labor. While these endocrinologic functions have been extensively documented, what may come as a surprise to many is that progesterone has *immunomodulatory* capabilities as well.

## 2. Immunosuppressive Capabilities of Progesterone

In 1977, Siiteri et al. [1] posed the rather provocative question of whether progesterone is “nature’s immunosuppressant”, based on early studies on the suppression, by progesterone, of some in vitro immune responses such as mixed-lymphocyte reactions and mitogen-stimulated proliferation of lymphocytes. The authors hypothesized that progesterone helps protect the conceptus from maternal immunologic rejection by suppressing maternal immune reactivity. Indeed, several decades ago progesterone was shown to be capable of delaying skin graft rejection in rats [2] and in sheep [3], while even earlier than that Black et al. [4] and Rowson et al. [5] described the effects of progesterone in inhibiting immune clearance of bacteria in the uteri of rabbits and cattle. These and other similar studies led to the suggestion that progesterone inhibits maternal immune reactions at the utero–placental interface [6].

More recent studies have helped refine our understanding of the inhibitory effects of progesterone on immune responses, particularly inflammatory responses. Progesterone inhibits the activation of murine dendritic cells [7], macrophages [8] and natural killer (NK) cells [9]. Treatment of lipopolysaccharide (LPS)-stimulated rat dendritic cells with progesterone suppresses the production of the pro-inflammatory cytokines tumor necrosis factor (TNF)-α and interleukin (IL)-1 β [10]. The secretion of the Th1-inducing cytokine IL-12 is also suppressed by progesterone [11]. Many of these inhibitory effects are mediated via suppression of NF-kB activation [12]. In addition to inhibiting the production of cytokines, progesterone has been reported to suppress the production of chemokines such as macrophage inflammatory protein-1α, macrophage inflammatory protein-1β and RANTES by CD8^+^ T lymphocytes [13].

Progesterone is capable of mediating interesting immunoregulatory roles by manipulating the generation of different immune cell types. For example, it induces the development of tolerogenic dendritic cells; and disruption of the interaction between progesterone and dendritic cells has been shown recently to result in poor generation of CD4^+^ T regulatory cells, and this is associated with poor placentation and intrauterine growth restriction in mice [14]. Many of the immunological effects of progesterone are brought about by a downstream mediator, the progesterone-induced blocking factor (PIBF). The importance of PIBF in immunoregulation during pregnancy is borne out by a recent study, which showed that decidual and peripheral NK activity are increased in PIBF-deficient mice, while T cell activation genes are downregulated in CD4^+^ T cells and upregulated in CD8+ T cells; moreover, T cells differentiate into Th1 cells. Interestingly, PIBF-deficient mice have lower implantation rates and higher rates of fetal loss as compared to mice with intact PIBF activity [15].

Thus, progesterone clearly has immunomodulatory (i.e., suppressive or inhibitory) effects on several immune responses (reviewed in [16]).

The genomic actions of progesterone are mediated by two intracellular receptors, progesterone receptor A (PR-A) and progesterone receptor B (PR-B) [17]; these two receptors are associated with different functions, with PR-A having roles in implantation and decidualization and PR-B needed for the development of the mammary gland [18]. Non-genomic actions of progesterone, such as uterine functions, are brought about by activating G protein-coupled progesterone receptors on cell membranes [19]. The immunological effects of progesterone, such as the suppression of T cell activation during pregnancy, are generally mediated via these progesterone receptors. Peripheral blood mononuclear cells and peripheral γδ T cells in pregnant women have been shown to have nuclear progesterone receptors [20], while circulating NK cells of pregnant women express both isoforms of progesterone receptors [21]. Decidual dendritic cells express progesterone receptors and are highly responsive to high local concentrations of progesterone [14]. Purified uterine NK cells do not express progesterone receptors [22], but interestingly enough their function is indeed affected by progesterone; it is suggested that progesterone acts on these cells via glucocorticoid receptors [23].

Progesterone is known to contribute significantly to the crosstalk between different cells in the uterus and placenta to affect different processes. Progesterone influences decidualization by controlling the differentiation of endometrial stromal cells [24], and disruption of this signaling can lead to pregnancy complications such as recurrent miscarriage and pre-eclampsia, emphasizing the importance of progesterone in this cellular crosstalk. Ovarian secretion of progesterone stimulates the production of activin A by endometrial cells, which influences the implantation of the trophoblast [25]. Thus, progesterone is known to be a critical player in inducing cellular changes that facilitate embryonic implantation and placental decidualization [26]. While such endocrinological roles of progesterone have been well documented previously, the contributions of progesterone to crosstalk with immune cells in the placenta have become clear in recent years.

For example, progesterone was shown to exert an immune tolerogenic effect by enhancing the phagocytic ability of trophoblasts and increasing trophoblast expression of anti-inflammatory mediators such as transforming growth factor (TGF) β [27]. In fact, Fujiwara suggested that the maternal immune system cooperates with the endocrine system in a systemic crosstalk to engineer the protection and growth of the embryo and that progesterone is a key player in this communication [28]. Verma and coworkers reported a decline in levels of myeloid-derived suppressor cells in women with early miscarriage, and this appeared to be associated with a significant decline in progesterone levels; interestingly, this was also associated with an increased bias towards Th1 cytokine production [29]. The crucial role of progesterone in immune crosstalk in the placenta was highlighted in a recent study, which showed that Brucella abortus infection in pregnant mice resulted in the suppression of progesterone production by the placenta, but that the administration of progesterone resulted in reduced production of inflammatory cytokines by trophoblast cells and reduced placental inflammation and increased viability of embryos [30]. These observations clearly highlight the important immunoregulatory roles played by progesterone at the level of the placenta.

## 3. Cytokines and Pregnancy Complications

While our immune system, with its exquisite specificity and amazing diversity of responses, is what stands between us and a bewildering variety of pathogens, it is unfortunately also responsible for strong adverse reactions such as hypersensitivities and autoimmune diseases. As far as the reproductive system is concerned, the immune system can also interfere with fertilization and cause autoimmune infertility [31,32], and pertinent to this review is the fact that the maternal immune system can also negatively impact pregnancy. In fact, pregnancy is not nearly as successful as laypersons generally assume; numerous potential complications may arise during the long period of gestation from fertilization to parturition. These include complications such as spontaneous miscarriage, pre-eclampsia and preterm delivery, and a huge body of literature indicates that the maternal immune system can and does contribute to these conditions.

A great deal of attention has been directed at investigating maternal cell-mediated immune effectors as possible etiologic factors for pregnancy complications; these include T lymphocytes, macrophages and NK cells in maternal peripheral blood and in uteroplacental tissues. The revolutionary discovery of the different subsets of T helper (Th) lymphocytes and the cytokines produced by them has led to deeper insights into maternal–fetal immunology. Cytokines, as crucial effectors of cell-mediated immunity, have justifiably received a great deal of attention.

How are cytokines relevant to pregnancy and to pregnancy loss? Cytokines are critical, indispensable mediatory molecules in the immune system; they play vital signaling roles primarily between a whole variety of cells of the immune system, and also between other cells in the body. Cytokines mediate a remarkable array of immune responses such as the stimulation of humoral and cell-mediated beneficial immune responses to infections [33]; however, cytokines also mediate autoimmune reactions that result in autoimmune diseases [34], hypersensitivity reactions [35] and inflammatory processes that lead to tissue damage [36]. Considering their pluripotent and powerful actions, it is not surprising that cytokines synthesized and secreted by immune cells such as T helper (Th) lymphocytes, macrophages and NK cells have been investigated in the context of the maternal–fetal relationship. Th1 and Th2 cells are the major subsets of Th cells, with different characteristic profiles of cytokine production and thus different roles in immune responses [37,38,39]. The Th1 subset of cells secretes the cytokines interferon (IFN)-γ, tumor necrosis factor (TNF)-β, TNF-α and interleukin (IL)-2, the very cytokines that instigate and sustain strong cell-mediated and inflammatory reactions such as cytotoxicity and delayed-type hypersensitivity. These inflammatory cytokines are implicated in graft rejection reactions, autoimmune disease pathology and inflammatory tissue damage. Th2 cells, on the other hand, secrete the cytokines IL-4, IL-5, IL-6, IL-10 and IL-13, which stimulate antibody production. Th1 and Th2 cells are mutually antagonistic to each other; some Th1 cytokines suppress the activation and/or the function of Th2 cells and vice versa [37,38,39].

### 3.1. Recurrent Spontaneous Miscarriage

Miscarriage is defined as the spontaneous loss of a fetus before the 20th week of pregnancy, while recurrent spontaneous miscarriage (RSM) is defined as two or more consecutive pregnancy losses. In some countries RSM is defined on the basis of three or more miscarriages; however, institutions such as the American Society of Reproductive Medicine and the American College of Obstetrics and Gynecology define RSM as two or more consecutive losses.

Animal experiments have shown that cellular immunity mediated by effector cells [40,41] and/or cytokines [42,43,44] released by them are detrimental to the conceptus. Single low doses of the inflammatory Th1 cytokines TNF-α, IFN-γ and IL-2 into pregnant mice cause abortions, while anti-TNF-α antibodies reduce abortion rates in a murine model of natural, immunologically mediated abortion [42]. The pro-inflammatory cytokines TNF-α and IFN-γ inhibit the outgrowth of human trophoblast cells in vitro [43] and synergistically provoke the apoptosis of human primary villous trophoblast cells [44]. Given that pro-inflammatory or Th1 cytokines have potent cytotoxic and tissue-damaging effects [34,35,36], as well as anti-pregnancy capabilities, it is not unexpected that unexplained recurrent spontaneous miscarriage (RSM) is associated with a greater bias toward Th1 or pro-inflammatory cytokines (reviewed in [45]).

A noteworthy study by Hill and colleagues [46] showed that peripheral blood cells from women with a history of RSM stimulated with human trophoblast antigens produced much higher levels of Th1 cytokines with embryotoxic activity. We subsequently demonstrated that mitogen-stimulated peripheral lymphocytes from women with unexplained RSM produce significantly elevated levels of the pro-inflammatory cytokines IL-2, IFN-γ and TNF-α, while on the contrary, women with a history of healthy pregnancy produce significantly greater levels of the anti-inflammatory Th2 cytokines IL-4, IL-5 and IL-10 [47,48]. This was confirmed by investigating maternal immune reactivity to placental antigens by stimulating maternal peripheral blood lymphocytes with either autologous placental cells or a trophoblast antigen preparation [49]. Ratios of pro-inflammatory cytokines to anti-inflammatory cytokines were higher in women who had a history of RSM as compared with women with a history of healthy pregnancy, substantiating the contention of an association between dominance of Th1 cytokines with RSM as opposed to a stronger Th2 cytokine dominance in healthy pregnancy [50]. Even before any upcoming pathology could be detected, increased production of IFN-γ and IL-2 and decreased production of the anti-inflammatory cytokines IL-4 and IL-10 were detected in women who eventually had a spontaneous miscarriage [51].

Investigations on cytokine profiles at the maternal–fetal interface have shown similarities with the cytokine milieu in the peripheral blood. Lower numbers of T cell clones producing anti-inflammatory cytokines were found in the decidua of women with unexplained RSM than in the decidua of women undergoing healthy pregnancy [52]. Endometrial expression of pro-inflammatory cytokines is higher, while that of anti-inflammatory cytokines is lower in women with idiopathic recurrent miscarriage as compared to controls [53]. Thus, women with unexplained recurrent miscarriage have increased levels of Th1 cytokines, while women with healthy pregnancy have decreased levels of Th1 cytokines and increased levels Th2 cytokines. In other words, women with unexplained recurrent miscarriage have an increased pro-inflammatory cytokine bias [45,46,47,48,49,50,51,52,53]. This suggests that normal pregnancy is associated with a natural pregnancy-induced modulation of maternal immune reactivity with a downregulation of Th1 responses and upregulation of Th2 responses.

### 3.2. Pre-Eclampsia

Pre-eclampsia (PE) is a common and dangerous multisystem complication of pregnancy, associated with increased blood pressure and proteinuria and a high proportion of maternal and infant deaths. Pre-eclampsia is generally defined as the occurrence of new-onset hypertension and proteinuria or other end-organ damage occurring after 20 weeks of gestation. PE is the most common hypertensive disorder of pregnancy, affecting 2–10% of pregnant women [54] and causing 15–20% of maternal deaths worldwide [55].

In normal pregnancy, placentation involves structural alterations as well as adaptations of the maternal blood vessels essential to receive the blood flow needed by the developing fetus. The spiral arteries that open into the intervillous space develop into large vessels from the original small muscular arteries to accommodate the massive requirements of blood needed by the placenta. Placentas from women with PE show clear evidence of placental hypoperfusion and ischaemia, and placental spiral vessels show hyperplasia, arteriosclerosis, mural thrombi and fibrinoid necrosis [56]. The abnormally narrow spiral arteries lead to uterine hypoperfusion and abnormally high velocity of blood flow [57]. This is generally described as the early “placental” or “fetal” syndrome of PE; there is also a wide range of effects in the mother which comprise the late-stage “maternal” syndrome.

PE consists of a generalized dysfunction of the maternal endothelium; this includes endothelial lesions in various organs, perivascular edema, hemorrhage, small-vessel thrombosis and glomerular endotheliosis (reviewed in [58]). This widespread maternal endothelial damage appears to result from an exaggerated systemic inflammatory response that involves maternal leukocytes and proinflammatory cytokines. A significant upregulation of pro-inflammatory cytokines such as TNF-α [59], IL-1 [60], IL-2 [61] and IL-18 [62] has been shown in pre-eclamptic placentas. IL-18 is a proinflammatory cytokine, which, in the presence of IL-12, skews immune reactivity towards a Th1 phenotype. High levels of IL-18 along with high levels of IL-12 in PE have been proposed to cause Th1 dominance [63]. Increased production of IFN-γ, a Th1 proinflammatory cytokine, has been found in placenta from pre-eclamptic pregnancies [64]. PE is also associated with decreased placental production of the anti-inflammatory cytokine IL-10 [59,65].

Sera from women with PE have been shown to have increased Th1/Th2 cytokine ratios, supporting the existence of a systemic pro-inflammatory condition in PE [66]. Peripheral blood mononuclear cells (PBMC) from pre-eclamptic women produce higher levels of the pro-inflammatory cytokines TNF-α [67], IFN-γ [68], IL-2 [69] and IL-1 [70]. On the other hand, reduced production of IL-4 [71], IL-10 [72,73] and IL-5 [74] by PBMC from patients with PE has been reported. We have demonstrated significantly increased production of the pro-inflammatory cytokines IFN-γ and TNF-α by women with PE versus women with normal pregnancies, who on the contrary showed significantly greater production of the Th2 cytokines IL-4, IL-5 and IL-10. A comparison of the ratios of Th2 to Th1 cytokines indicated significantly higher Th1/pro-inflammatory cytokine production in PE as compared to normal pregnancies [75]. Thus, there is clear evidence to indicate an increased dominance of Th1 or pro-inflammatory cytokines in women with pre-eclampsia, both at the maternal–fetal interface and in the periphery (reviewed in [76]).

### 3.3. Preterm Delivery

Preterm labor, defined as labor that starts before 37 weeks of gestation, and preterm delivery, defined as any birth before 37 weeks of gestation, occur in about 12% of pregnancies. Preterm delivery (PTD) is a major cause of perinatal morbidity and mortality and is an important complication to be overcome in the field of obstetrics [77]. Preterm labor (PTL) is suggested to be instigated by precocious activation of elements that normally initiate delivery at term.

Inflammation in the upper genital tract plays a major role in the pathogenesis of preterm labor [78], and by corollary, this implicates causative roles for immunological effectors such as cytokines. We have demonstrated an immune deviation towards Th1 cytokine bias in a proportion of women with PTD [79]; PBMC from women with normal pregnancy produce higher levels of the Th2 cytokines IL-4, IL-5 and IL-10, while the Th1 cytokines IL-2 and IFN-γ are produced at greater concentrations by women with PTD. Furthermore, the ratios of type 1 to type 2 cytokines are indicative of a bias towards stronger Th1 reactivity in PTD. Several studies have demonstrated increased serum levels of pro-inflammatory cytokines such as TNF-α, IL-1, IL-6, IL-8 and IL-12 in women with preterm birth as compared to term birth [80,81,82,83]. Evidence has also been provided of higher levels of inflammatory cytokines in uterine tissues (reviewed in [83]), cervicovaginal fluid [84] and in the placenta [85,86] of women with PTD. These observations suggest an overall association of some Th1 cytokines with preterm labor.

Preterm labor in the setting of infection has been proposed to develop from the actions of proinflammatory cytokines secreted as part of the fetal and/or maternal host response to microbial invasion [87]. Dudley proposes that in addition to culture-proven infection, a so called “intrauterine inflammatory response syndrome” may be responsible for preterm labor in which no infectious organisms are identified; in fact, high levels of inflammatory cytokines may be a mechanism that could form the pathophysiologic basis for this association [88].

We have presented a summary of the immunosuppressive properties of progesterone, and evidence that points to an association between maternal Th1-mediated immune responses and pregnancy complications such as recurrent spontaneous miscarriage, preeclampsia and preterm delivery. We will proceed to discuss the relevance of progesterone to therapeutic immunomodulation of Th1 cytokine responses.

## 4. Supplementation with Progestogens

A landmark study showed that progesterone suppresses the production of Th1 cytokines by trophoblast antigen-activated peripheral blood cells from women with unexplained RSM [89]. Progesterone also facilitates the development of Th2 cells in vitro, leading to the inference that progesterone promotes fetal survival by inducing the production of Th2 cytokines [90]. Pro-inflammatory cytokines are associated with recurrent pregnancy loss on the one hand [42,43,44,45,46,47,48,49,50,51,52,53] and on the other, progesterone has potentially very useful immunomodulatory capabilities [7,8,9,10,11,12,13,14,15,16,20,21,22,23,27,28,29,30,89,90]; this has led to the exploration of progesterone supplementation to treat pregnancy complications.

### 4.1. Supplementation with Progesterone

Progesterone has been proposed for use in three pregnancy complications: recurrent miscarriage, premature labor/birth and pre-eclampsia [91]. A meta-analysis in 2003 of 14 trials with 1988 women revealed no statistically significant difference in the risk of miscarriage between progestogen and placebo or no-treatment groups, when all women, regardless of gravidity and number of previous miscarriages, were included in the meta-analysis [92]. However, an update by Haas and Ramsey [93] in 2008 indicated that there was evidence of a beneficial treatment for women with a history of recurrent miscarriage. The authors concluded that progesterone supplementation may be warranted based on the reduced rates of miscarriage in women who received progesterone. A critical evaluation of randomized, placebo-controlled trials concluded that vaginal micronized progesterone results in increased live birth rates, and that women with recurrent miscarriage who present with bleeding in early pregnancy may benefit from the use of vaginal micronized progesterone [94]. A meta-analysis of 12 trials comprising 1856 women led Haas and colleagues to conclude that there may be a reduction in the number of miscarriages for women who received progesterone versus those who received a placebo [95]. Thus, there is evidence for progesterone supplementation leading to higher rates of live birth and ongoing pregnancy (Table 1) (reviewed in [96]). However, some studies have failed to demonstrate a significant beneficial effect in recurrent miscarriage. A randomized double-blind, placebo-controlled study on women with unexplained recurrent miscarriage who received micronized progesterone in the first trimester found no evidence for improved live-birth rates as compared to women who received a placebo [97]. The lack of consistent data supporting a strong beneficial effect of progesterone supplementation in recurrent miscarriage is likely due to studies having been carried out on unselected populations with recurrent miscarriage. Carp suggests that immunotherapy can be effective “when the population is selected for a poor prognosis, or immune phenomena” [98]. 

Progesterone supplementation shows promise in preventing preterm labor. A while ago this was believed to be ineffective, but recent studies indicate its benefits, a fact that Schmouder and colleagues [99] refer to as the “rebirth of progesterone in the prevention of preterm labour”. An analysis of randomized, double-blind, placebo-controlled trials led these authors to affirm that progesterone is both effective and safe in reducing the risk of preterm birth in women who had previous preterm births [99]. An earlier analysis of seven randomized controlled trials concluded that women who received progesterone were significantly less prone to preterm delivery [100]. Matie et al. [101] concluded that only very few interventions are effective in preventing preterm birth, and progesterone supplementation is one of them.

### 4.2. Supplementation with the Oral Progestogen Dydrogesterone

Orally administered progestogen, dydrogesterone (6-dehydro-9β, 10α-progesterone) (Duphaston^®^, Abbott Laboratories, Abbott Park, IL, USA), has received well-deserved attention in this context. It is used widely to treat menstrual disorders, luteal insufficiency, threatened abortion and in hormone replacement therapy. Dydrogesterone is similar to endogenous progesterone in terms of molecular structure and pharmacological effects, but is more potent than natural progesterone, with a higher affinity for the progesterone receptor than progesterone itself [102,103]. Dydrogesterone has also been shown to have more bioavailability than progesterone [104].

The culture of peripheral blood mononuclear cells (PBMC) from women with unexplained spontaneous recurrent miscarriage in the presence of dydrogesterone results in the production significantly decreased levels of the Th1 (pro-inflammatory) cytokines IFN-γ and TNF-α and significantly elevated levels of the Th2 cytokines IL-4 and IL-6 [105]. Th1/Th2 cytokine ratios are thus significantly reduced when PBMC are exposed to dydrogesterone, indicating a decrease in Th1 or pro-inflammatory cytokine bias. The progesterone receptor antagonist RU486 inhibits the cytokine-modulating actions of dydrogesterone, which indicates that these actions are mediated via the progesterone receptor [105]. In addition to suppressing the secretion of TNF-α and IFN-γ, dydrogesterone also inhibits the production of cytokine IL-17 [106], a potent pro-inflammatory and chemotactic cytokine. Indeed, IL-17 has been associated with embryonic loss in animal studies and with miscarriage in humans. The injection of IL-17 into pregnant mice has been shown to result in embryonic loss [107]. Elevated levels of IL-17 have been reported in the peripheral blood and decidua of women with recurrent pregnancy loss [108], and the incidence of unexplained RSM is associated with increased levels of serum IL-17 and Th17/Treg cell ratios in peripheral blood and at the maternal–fetal interface [109]. Thus, dydrogesterone has potent immunomodulatory properties demonstrated as suppression of pro-inflammatory cytokine production in vitro [110].

The ability of dydrogesterone to downregulate cytokines that are detrimental to pregnancy has been proposed to be conducive to healthy pregnancy [110]; indeed, supplementation with dydrogesterone has been shown to be beneficial in recurrent miscarriage. An early study by El-Zibdeh showed that dydrogesterone-treated women with unexplained RSM had fewer miscarriages compared with women given a placebo [111]. A prospective, open, randomized study to ascertain whether dydrogesterone prevents miscarriage in women with vaginal bleeding up to 16 weeks of pregnancy showed that miscarriage occurred in 12.5% of women treated with dydrogesterone as compared to 18.4% of women with conservative management [112]. A randomized, double-blind, placebo-controlled study on dydrogesterone supplementation by Kumar et al. [113] demonstrated a significant decrease in the number of miscarriages as well as an increase in the mean gestational age at delivery.

Carp’s meta-analysis in 2015 concluded that there was a 10.5% miscarriage rate in women who received dydrogesterone as compared to 23.5% in control women who did not; this analysis showed significant reduction of 29% in the odds for miscarriage indicating a real treatment effect [114]. A recent meta-analysis on 13 studies comprising a total of 2454 patients concluded that the pregnancy success rate in women treated with dydrogesterone was significantly higher [115]. Saccone et al.’s systematic review and meta-analysis of ten randomized controlled trials indicated that supplementation with dydrogesterone reduces the rate of miscarriage [116] (Table 1).

**Table 1:** Summary of studies on supplementation with progesterone and dydrogesterone.

Ref. No.	Type of Study	Outcome of Study
**Supplementation with Progesterone**		
[92]	Meta-analysis of 14 trials (2003)	No difference in risk of miscarriage
[93]	Update of above study (2008)	Reduced rate of miscarriage
[94]	Critical evaluation of randomized, placebo-controlled trials	Increase in live-birth rate
[95]	Meta-analysis of 12 trials	Reduction in number of miscarriages as compared to placebo
[97]	Randomized, placebo-controlled study on women with uRSM *	No evidence of improved live-birth rate
**Supplementation with Progesterone**		
[111]	Placebo-controlled study on dydrogesterone supplementation in women with uRSM	Fewer miscarriages as compared to placebo
[112]	Prospective, open, randomized study on women with uRSM	Significant reduction in miscarriage
[113]	Randomized, double-blind, placebo-controlled study	Significant decrease in number of miscarriages, increase in mean gestational age at delivery
[114]	Meta-analyses of studies on dydrogesterone supplementation	Significant reduction in odds for miscarriage
[115]	Meta-analysis of 13 studies on dydrogesterone supplementation	Significantly higher pregnancy rate
[116]	Systemic review and meta-analysis	Significant reduction in rate of miscarriage

* uRSM, unexplained recurrent miscarriage.

Schindler avers that in addition to the use of dydrogesterone in recurrent miscarriage, it may also be considered for use in preventing or treating other pregnancy disorders such as preterm labor and preeclampsia [117]. In fact, based on its demonstrated ability to reduce the development of pre-eclampsia, it is proposed that it can be considered for use in pre-eclampsia and to continue it until late pregnancy to prevent premature labor as well [118]. The ability of dydrogesterone to increase the production of IL-10 and PIBF (discussed below) and to decrease the production of IFN-γ, make it worth considering for treating pre-eclampsia [119].

A pilot study on women with higher risk of developing gestational hypertension showed that those who received dydrogesterone had a significantly lower incidence as compared to those who did not (2% vs. 13%) [120]. Women with high risk factors of pre-eclampsia on dydrogesterone developed significantly fewer disorders such as hypertension, proteinuria fetal growth retardation syndrome, preterm labor and also had significantly reduced incidence of pre-eclampsia [121]. A retrospective comparative analysis on pregnancies that followed assisted reproductive technique showed that the incidence of pre-eclampsia was lower in those who received dydrogesterone [122].

More data from large trials on the usefulness of dydrogesterone supplementation in preterm labor and pre-eclampsia are clearly needed, but the available data give scope for optimism.

The advantages of dydrogesterone from the perspective of safety are that it does not inhibit ovulation at the recommended doses, it is devoid of estrogenic or androgenic properties and it does not cause metabolic side effects [123]. The safety and tolerability of dydrogesterone treatment are well established, and the benefit–risk profile is reported to be favorable [124]. In fact, dydrogesterone offers some advantages over micronized progesterone, which is absorbed poorly, has a short biologic half-life [125] and is cleared quickly [126]. Another significant advantage is that dydrogesterone retains its immunomodulatory activity even after it is converted to its major metabolite after oral administration [127].

## 5. Progesterone-Induced Blocking Factor (PIBF)

A protein first described 36 years ago has turned out to have fascinating and pertinent roles in the success of pregnancy [128]. This molecule, the progesterone-induced blocking factor (PIBF), has several immunomodulatory capabilities; the most relevant in the context of this review is its ability to induce a Th2-biased status. PIBF binds to PIBF receptors after which the receptor forms a heterodimer with the IL-4 receptor and activates the Jak1/Stat6 pathway [129], leading to increased production of Th2 type cytokines. This contributes to the Th2-dominant cytokine pattern that is maintained during normal pregnancy. Spleen cells from non-pregnant female mice when treated with PIBF produce significantly higher levels of IL-4 and IL-10 [130]. Similarly, lymphocytes from women with recurrent miscarriage and women with preterm delivery when exposed to PIBF produce lower levels of Thl-type cytokines and increased levels of Th2-type cytokines [131].

PIBF appears to mediate its critical role in pregnancy in very interesting ways. Depletion of PIBF in mice during early pregnancy results in impaired implantation of embryos and increased resorption rates in mice, along with increased decidual and peripheral NK activity [15]. The blocking of PIBF with anti-PIBF antibodies, or the inhibition of PIBF synthesis, results in Th1-dominant cytokine production, significantly increased NK activity and fetal loss, which is corrected by the treatment with anti-NK antibodies [132]. PIBF treatment in a rat model of pre-eclampsia normalizes the Th1/Th2 ratio, reduces the inflammation, corrects the blood pressure and prevents fetal growth retardation [133]. Interestingly, this study showed that levels of circulating and placental cytolytic NK cells and IL-17 were reduced significantly, while levels of IL-4 and Th2 cells were significantly increased in rats after PIBF administration. The authors concluded that these effects on blood pressure and inflammation were brought about by PIBF normalizing the levels of Th2 cells. Serum concentrations of PIBF in normal human pregnancy increase with gestational age; lower than normal concentrations are associated with spontaneous termination of pregnancy [134]; failure to detect PIBF at 3 to 5 weeks of gestation has been shown to be associated with a higher rate of miscarriage [135]. In fact, the decidual expression of PIBF and serum levels of PIBF in women with unexplained miscarriages are significantly lower than those in healthy pregnant women, pointing to important beneficial roles of PIBF [136].

Dydrogesterone supplementation in women undergoing threatened miscarriage proved to be beneficial, and interestingly, this was associated with increased levels of PIBF [137]. We suggest that one of the key pathways by which dydrogesterone mediates its immunomodulatory effects is via the stimulation of production of PIBF, which induces a Th2-dominant cytokine response, by facilitating the production of IL-4 and IL-10, thus altering the Th1/Th2 balance in favor of pregnancy [131]. The administration of dydrogesterone in women at risk of preterm labor resulted in increased production of both PIBF and Th2 cytokines, as well as decreased production of Th1 cytokines, suggesting that it could be considered for prevention or treatment of preterm labor and delivery [119].

**Figure 1:**
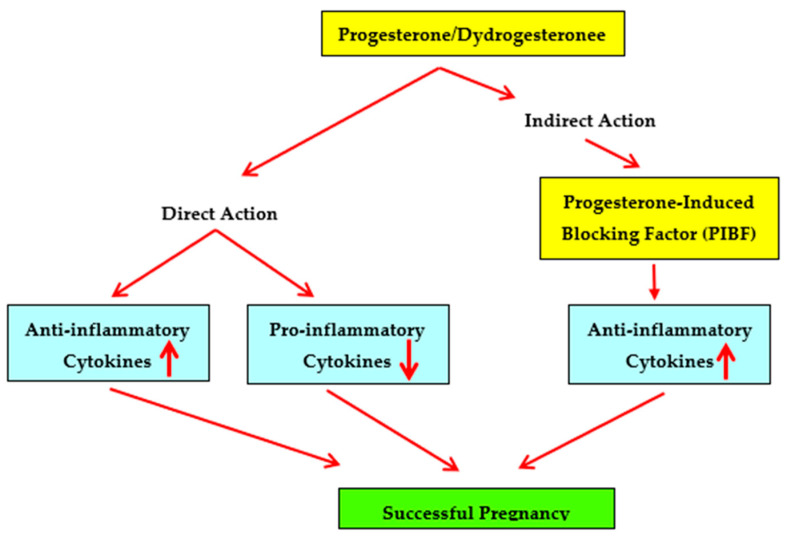
Immunomodulatory effects of progestogens on cytokine production patterns.

In summary, studies showing that dydrogesterone is an immunomodulator that shifts the maternal cytokine balance from a Th1 or pro-inflammatory bias towards a Th2 or anti-inflammatory bias and studies showing that dydrogesterone supplementation is beneficial in recurrent miscarriage suggest that dydrogesterone may be considered for effective, safe and orally administered therapy in unexplained recurrent spontaneous miscarriage (Figure 1).

## 6. Future Perspectives

While progesterone, dydrogesterone and PIBF show promise as immunomodulators that can be considered for use in preventing or treating pregnancy complications, more basic and clinical research is certainly warranted along several lines. The precise molecular pathways defining the mechanisms of action of progestogens and PIBF need to be completely worked out. Several studies and trials have been conducted on progestogen supplementation for unexplained recurrent miscarriage; but this needs to be characterized further by focusing on immunologically mediated recurrent spontaneous miscarriage, for example, in women with a predominantly Th1-biased status. This would entail a personalized medicine approach rather than a “one size fits all” approach, as immunomodulation is likely to work only on women with immune etiologies. As for other pregnancy complications, large clinical trials on progestogen supplementation in preterm labor and pre-eclampsia are awaited.

## Data Availability

Not applicable.

## Abbreviations

IFN	Interferon
IL	Interleukin
PBMC	Peripheral blood mononuclear cells
PE	Pre-eclampsia
PIBF	Progesterone-induced blocking factor
PR	Progesterone receptor
PTD	Preterm labor
RSM	Recurrent spontaneous miscarriage
Th	T helper
TNF	Tumor necrosis factor
